# Diagnosis of Severe Acute Respiratory Syndrome Coronavirus 2 Infection in a Patient Presenting With Guillain-Barré Syndrome

**DOI:** 10.7759/cureus.19147

**Published:** 2021-10-30

**Authors:** Joseph P McGann, Ronak Bahuva

**Affiliations:** 1 Emergency Medicine, University at Buffalo Jacobs School of Medicine and Biomedical Sciences, Buffalo, USA; 2 Internal Medicine, University at Buffalo Jacobs School of Medicine and Biomedical Sciences, Buffalo, USA

**Keywords:** nerve conduction study (ncs), post covid-19 complication, covid induced guillian barre syndrome, guillian barré syndrome, covid-19

## Abstract

Guillain-Barré syndrome (GBS) is a rare neurologic disorder in which the host immune system damages peripheral nerve cells. Although classically described as an ascending paralysis, GBS can present in a myriad of ways. As peripheral nerve conduction becomes impaired, symptoms can range from mild weakness to a life-threatening paralysis of the respiratory muscles. Patients frequently experience prolonged recovery times, with residual effects often lasting up to a year or longer. The exact cause of the disorder is not fully understood, but its development most often follows infection with numerous different pathogens. Most recently, severe acute respiratory syndrome coronavirus 2 (SARS-CoV-2) infection has been identified as a possible cause. We present the case of a 63-year-old male presenting with signs and symptoms consistent with GBS who was later identified to have recently been infected with the SARS-CoV-2. Our report adds to the growing number of GBS cases that have been associated with SARS-CoV-2 and prompts further consideration of the potential sequelae of COVID-19.

## Introduction

A newly identified virus of the coronaviridae family, severe acute respiratory syndrome coronavirus 2 (SARS-CoV-2), has caused a worldwide pandemic called COVID-19 [1]. The first reported cases of the disease came from the Wuhan province of China in December 2020, following which the World Health Organization (WHO) declared COVID-19 a pandemic on March 11, 2020 [2]. Since that time, the virus has spread aggressively throughout the entire globe. As data began to emerge regarding the pathogenesis and clinical manifestations of the disease, the lungs were initially considered the primary target organ for viral proliferation. However, COVID-19 is now known to be a multi-system disease, affecting numerous organ systems in the body, including the brain, heart, lungs, liver, kidneys, and intestines [3]. 

With the progression of the pandemic, several short- and long-term post-infectious complications are being identified, with the nervous system appearing especially vulnerable [4,5]. One such neurologic complication is Guillain-Barré syndrome (GBS), an acute inflammatory demyelinating polyneuropathy that typically presents as a rapidly progressing ascending paralysis of the lower extremities which can lead to a devastating and life-threatening paralysis [6,7]. The condition is thought to be caused by an autoimmune mechanism of molecular mimicry between a foreign protein and human peripheral nerve gangliosides, leading to the formation of antibodies against the ganglioside protein, and without early intervention with either intravenous immunoglobulins (IVIGs) or plasmapheresis, the paralysis can progress to involve the phrenic and cranial nerves, resulting in significant morbidity and mortality [6,7]. Prior to the COVID-19 pandemic, a majority of cases of GBS were reported within four weeks of a gastrointestinal tract or respiratory infection [6,7]. An increasing number of cases of GBS are now being reported in patients who have recovered from COVID-19, with the symptoms of GBS appearing after an average of 11.92 ± 6.20 days after the infection in majority of cases, with cases presenting as late as 90 days after infection [8,9]. In this case report, we describe the retrospective diagnosis of COVID-19 in a patient who presented with symptoms of GBS. 

## Case presentation

A 63-year-old Caucasian male with no significant past medical history presented to the emergency department with complaints of bilateral lower-extremity muscle weakness and paresthesia. The patient first noticed a tingling sensation and bilateral lower-extremity myalgias approximately two weeks prior to presentation beginning the morning after engaging in a vigorous snow shoveling session. He initially attributed the symptoms to his shoveling; however, he reported no traumatic events while shoveling and did not experience any symptoms immediately afterward. Over the next few days, the patient’s symptoms began spreading to his back and upper extremities, yet he was unable to describe any specific pattern of spread. The patient initially saw his primary care physician who started him on gabapentin for his neuropathic symptoms and encouraged him to follow up with outpatient neurology. Due to extenuating circumstances regarding pandemic-associated restrictions, the patient was unable to secure an outpatient neurology appointment date for more than a month out, prompting him to visit the emergency room.

Upon presentation, the patient reported occasional shock-like sensations in his fingertips, progressive difficulty with ambulation, and pain between his shoulder blades that had interfered with his ability to sleep. He was noted to have been very active leading up to his presentation and was unable to identify any precipitating factors for his illness aside from his shoveling. After extensive questioning for additional history, the patient reported a known COVID-19 exposure when his wife tested positive about four weeks prior to the onset of his current symptoms. He was never tested at that time but did experience fatigue, headaches, mild cough, and loss of taste. He completed a 14-day quarantine at home, and apart from the loss of taste, all his other symptoms had completely resolved. He saw no association between those symptoms and his current symptoms. 

On examination, the patient was in no acute distress with no evidence of respiratory compromise. Muscle strength was 4/5 bilaterally in the lower extremities and 5/5 in the upper extremities. No sensory abnormalities were noted. Deep tendon reflexes were absent bilaterally in the lower extremities and 1+ in the upper extremities. Gait was grossly abnormal and unsteady. The physical examination was otherwise unremarkable. The patient denied any difficulty breathing or bladder/bowel incontinence. Vital signs were all within normal limits, and the patient remained hemodynamically stable throughout his hospital course. Initial laboratory workup was significant for an elevated creatinine kinase of 472 units/L. Results of a complete blood count, comprehensive metabolic panel, lactate, vitamin B12 level, and C-reactive protein were otherwise within normal limits. Chest x-ray revealed no acute cardiopulmonary process, and brain and complete spinal MRI results were notable for only mild lumbar spinal stenosis and age-related degenerative changes. Lumbar puncture was performed under fluoroscopic guidance, and cerebrospinal fluid (CSF) analysis revealed albuminocytologic dissociation (Table 1). 

**Table 1:** CSF analysis Elevated CSF protein level without accompanying elevation in WBCs is consistent with pattern of albuminocytologic dissociation often seen in Guillain-Barré syndrome. CSF, cerebrospinal fluid; WBCs, white blood cells.

	Result	Reference Range
CSF Appearance	Clear	Clear
CSF Color	Colorless	Colorless
WBCs (cells/mm^3^)	4	0-5
Glucose (mg/dL)	61	40-70
Protein (mg/dL)	105	15-45

Reverse transcription polymerase chain reaction (RT-PCR) test was positive for SARS-CoV-2 antigen despite the patient having no symptoms of active disease, which likely represented residual virus from infection during the previous month. The clinical picture and albuminocytologic dissociation in CSF analysis were at this point consistent with GBS, likely from the recent COVID-19 infection as no other possible etiologic causes could be identified. Electromyography analysis was inconclusive to distinguish between axonal loss and demyelination; however, findings were consistent with a diffuse, largely symmetrical sensorimotor peripheral neuropathy of moderate degree. A “sural sparing” pattern was identified in the left lower extremity, which can sometimes be seen in the acute inflammatory demyelinating polyneuropathy subtype of GBS (Table 2) [10].

**Table 2:** Sensory nerve conduction studies Preserved SNAP in left sural nerve with diminished and absent SNAP values in left median and left radial nerves, respectively, is consistent with a “sural sparing” pattern of demyelination. SNAP, sensory nerve action potential.

	Result	Reference Range
Left Sural Nerve SNAP (mV)	6.3	>6
Left Median Nerve SNAP (mV)	14.6	>20
Left Radial Nerve SNAP (mV)	Absent	>10

The patient was started on a five-day course of 0.4 g/kg IVIG, as per neurology recommendations. He remained stable throughout treatment with excellent negative inspiratory forces. At the completion of treatment, he was medically cleared for a safe discharge to home, with recommendation to continue home physical therapy, and was instructed to follow up with neurology on an outpatient basis in 3-4 weeks. 

## Discussion

We report the case of a 63-year-old male who presented with signs and symptoms consistent with GBS after a likely COVID-19 infection in the preceding month. The diagnosis of GBS was confirmed with a pattern of albuminocytologic dissociation in CSF on lumbar puncture and sural nerve sparing pattern on nerve conduction studies, and the patient was successfully treated with IVIG. The patient did not initially report a history suggestive of the typical gastrointestinal or respiratory infections associated with GBS; however, further questioning revealed that he had experienced constitutional symptoms of COVID-19 after a known exposure to the virus. These symptoms had since largely resolved, yet an RT-PCR test detected the SARS-CoV-2 antigen with a high cycle threshold. This weakly positive test with low nucleic acid content is likely indicative of persistent positive PCR test from the recent infection, a phenomenon that has been described in the literature [11].

It is noteworthy that the clinical features, diagnostic findings, and response to standard treatment in our case did not appear to differ from previously reported cases of GBS associated with COVID-19 or other etiologies [9]. The novelty of our case is that we diagnosed COVID-19 retrospectively after the diagnosis of GBS from the patient’s history and the SARS-CoV-2 PCR test. As many patients with mild symptoms of COVID-19 do not get tested for COVID-19 infection, it is important to obtain an extensive history of possible COVID-19 infection or exposure from any patient presenting with signs and symptoms concerning for GBS. Testing for prior exposure to COVID-19 with RT-PCR and antibodies to SARS-CoV-2 should be considered to support the diagnosis of GBS in conjunction with more invasive diagnostic approaches such as lumbar puncture. Further research is required to identify any variations in patients with GBS associated with COVID-19 as compared to patients who develop the disease after infection with other known causative agents.

## Conclusions

GBS is a life-threatening neurologic disorder that often requires intensive intervention and prolonged rehabilitation. While the disorder is most commonly associated with *Campylobacter jejuni* infection in the United States, a number of different respiratory and gastrointestinal pathogens have been reported to cause GBS. We report a case of GBS that appears to have been triggered by infection with the SARS-CoV-2 virus. This adds to a growing number of cases that have been identified among individuals testing positive for COVID-19. While the full impact of the disease is not yet fully understood, the development of GBS should be considered as one of the many potential sequelae of COVID-19.
